# Differential response of Angus-Hereford and Rarámuri Criollo cattle to a dynamic feeding challenge during the training to an autonomous virtual fencing collar

**DOI:** 10.1093/jas/skag024

**Published:** 2026-02-03

**Authors:** Andrés R Perea, Lara K Macon, Maximiliano J Spetter, Micah P Funk, Mehmet Bakir, Richard E Estell, Brandon T Bestelmeyer, Andres F Cibils, Santiago A Utsumi

**Affiliations:** Department of Animal and Range Sciences, New Mexico State University, 2980 South Espina St., Las Cruces, New Mexico, 88003-8003, United States; Jornada Experimental Range, Agricultural Research Service, United States Department of Agriculture, 2995 Knox St., Las Cruces, New Mexico, 88003-8003, United States; Department of Animal and Range Sciences, New Mexico State University, 2980 South Espina St., Las Cruces, New Mexico, 88003-8003, United States; Department of Animal and Range Sciences, New Mexico State University, 2980 South Espina St., Las Cruces, New Mexico, 88003-8003, United States; Department of Animal and Range Sciences, New Mexico State University, 2980 South Espina St., Las Cruces, New Mexico, 88003-8003, United States; Jornada Experimental Range, Agricultural Research Service, United States Department of Agriculture, 2995 Knox St., Las Cruces, New Mexico, 88003-8003, United States; Jornada Experimental Range, Agricultural Research Service, United States Department of Agriculture, 2995 Knox St., Las Cruces, New Mexico, 88003-8003, United States; Oklahoma and Central Plains Agricultural Research Center, Agricultural Research Service, United States Department of Agriculture, 7207 W Cheyenne St., El Reno, Oklahoma, 73036, United States; Department of Animal and Range Sciences, New Mexico State University, 2980 South Espina St., Las Cruces, New Mexico, 88003-8003, United States

**Keywords:** Angus-Hereford, conditioning learning, precision livestock farming, Rarámuri Criollo, training, virtual fencing

## Abstract

Virtual fencing (VF) is an emerging concept for managing livestock distribution using smart-tracking collars. The collars apply Global Navigation Satellite System technology to emit sound alerts that warn animals of virtual boundaries enforced by electric pulses. Behavioral differences may explain how individuals and breeds respond to this technology. This work compared behavioral responses of non-lactating Rarámuri Criollo (RC) and Angus-Hereford (AH) cattle to a commercial VF system during the training phase. Thirty RC and 30 AH cows naïve to VF were fitted with Nofence collars and assigned by breed to rectangular pens (n = 3 per breed) in a completely randomized design. Wheat hay was provided ad libitum at feeding stations located on the east and west ends of each pen, which were made accessible or restricted via VF schedules applied across six 3-day periods. Period 1 had no restrictions; periods 2 and 3 restricted access to the west and east feeding stations, respectively; and periods 4–6 repeated these configurations. Behavioral responses, including number of auditory warnings, electric pulses, electric pulses to auditory warnings ratio, animal activity, and spatial distribution in pens, were evaluated using repeated measures mixed model ANOVA (α  =  0.05). AH cows received more auditory warnings and electric pulses on day 1 of period 2 and a greater ratio of electric pulses to auditory warnings throughout the study. RC cows spent more time within the designated VF containment zone on day 1 of period 2. AH cows exhibited consistently greater movement activity throughout the study. Overall VF containment was 97%, indicating that both breeds successfully learned and adapted to shifting virtual boundaries. These results suggest that breed-specific behavioral traits, including vigilance, risk assessment, feeding motivation, and activity, may underline differential responses to VF during early training.

## Teaser

Angus-Hereford and Rarámuri Criollo cattle were trained to a virtual fencing system using a dynamic feed challenge. Cattle breeds differ in how they learn and respond to virtual fencing cues; understanding these behavioral differences can help ranchers optimize virtual fencing training and improve livestock distribution on rangelands.

## Introduction

Virtual fencing (VF) is emerging as a promising precision livestock management technology for rangelands (Anderson 2007; Mayer et al. 2025). VF implements Global Navigation Satellite System (GNSS) technology to track animal locations and enforce virtual boundaries using auditory warnings associated to electric pulse reinforcements (Goliński et al. 2022). Briefly, the collar microcontroller calculates animal location, trajectory, and speed in relation to a user-defined virtual boundary; if the animal approaches the boundary, an auditory warning signal is triggered. If the animal fails to stop or alter its trajectory, then a mild electric pulse is reinforced (Anderson 2007; Goliński et al. 2022). By conditioning animals to associate auditory warnings with boundaries, VF offers a flexible alternative to physical fencing, enabling managers to guide livestock distribution dynamically while reducing infrastructure costs and labor (Anderson 2007).

In VF applications, animal containment or exclusion relies on the principle of conditioning learning (Skinner 1971), with associative learning between the auditory warnings and electric pulses serving as the underlying response mechanism (Lee et al. 2009). According to this principle, behaviors that are associated with a “satisfying” or rewarding outcome are likely to reoccur, whereas behaviors that are associated with a “discouraging” or unfavorable outcome are likely to decrease. Thus, animals associate distinguishable choices (eg, trespassing auditory boundaries) with an outcome (eg, receiving a mild electric pulse) and use encoded information to alter movements. Most commercial VF systems consist of solar-powered neck collars relying on advanced communication and network technology to control fences remotely (Goliński et al. 2022). The collars also allow real-time animal tracking (Hamidi et al. 2023). The use of dynamic fencing schedules offers opportunities for flexible grazing management (Umstatter 2011; Anderson et al. 2014).

The effectiveness of VF depends on how readily an animal learns to respond to auditory warnings without the need for electric pulse reinforcement. Training is therefore a critical step to implement this tool, but studies have reported variation in protocols, duration, and animal responses (Anderson 2007; Colusso et al. 2020; Keshavarzi et al. 2020; Nyamuryekung’e et al. 2023).

There is no consensus on VF training protocols. Some studies emphasize the role of individual learning, while others focus on effects of social facilitation among individuals. Campbell et al. (2018) trained heifers individually, using a fixed fencing enclosure applied on a small pen. Fay et al. (1989) enclosed goats in a small plot fenced with a static virtual boundary. Boyd et al. (2023) trained a group of 39 cows in a pen using a two-stage expansive auditory warning zone. Nyamuryekung’e et al. (2023) trained groups of 11 and 17 Brangus cows to a static enclosure established in a training corral. In studies conducted by Keshavarzi et al. (2020), Verdon et al. (2021), Aaser et al. (2022), Hamidi et al. (2022), and Campa Madrid et al. (2025), a variable-pressure VF was placed near existing physical or electric fence lines to train cattle on pasture. In these studies, the response of animals to VF was rarely uniform, underscoring the importance of learning as a self-regulated process (Provenza et al. 2003).

There is also no clear agreement on the optimal duration of VF training protocols, which can vary between 3 days (Campbell et al. 2018), 5 days (Fay et al. 1989), 12 days (Nyamuryekung’e et al. 2023), and up to 16 days (Fuchs et al. 2025). Lee and Campbell (2021) partitioned the learning process into three progressive phases. The initial phase starts when naïve animals experience a collar enforced electric pulse for the first time, typically exhibiting an acute stress response. During the second phase, animals begin to associate the auditory warning with the subsequent occurrence of an electric pulse. In the final phase, trained animals anticipate the occurrence and duration of cues, responding promptly to the collar auditory warnings while avoiding electric pulses. Ultimately, the low ratio of electric pulses to auditory warnings indicates effective learning and an ability to adapt to static or shifting VF boundaries.

Breed-specific traits could influence VF learning outcomes, yet comparative evaluations across cattle breeds remain limited. Rarámuri Criollo (RC), a heritage cattle biotype from northern Mexico (Estell 2021), are characterized by a calm temperament, vigilant mothering style, and plastic foraging behavior (Cibils et al. 2023). These traits, likely shaped by their unique genotype (Spetter et al. 2025) and centuries of natural selection (Anderson et al. 2015) make them well suited to the harsh climatic and nutritional conditions of the southwest US. In contrast, Angus-Hereford (AH) cattle, which are the most common commercial cattle in the US, often exhibit more consistent grazing patterns (Peinetti et al. 2011; Spiegal et al. 2019), lower mobility (Duni et al. 2023), and lower use of steeper slopes (Roacho Estrada et al. 2023). The RC cattle also have a smaller frame size and greater heat tolerance, adding to their suitability in hot and dry desert rangelands (Nyamuryekung’e et al. 2021; McIntosh et al. 2023). Given their behavioral and adaptive characteristics, RC cattle are well suited for VF applications in the southwest US (Campa Madrid et al. 2025). However, no direct comparison with more traditional AH cattle has been made previously.

The goal of this study was to compare how AH and RC cattle learn and adapt to a commercial VF system during controlled training trials. Four hypotheses were considered: 1) AH and RC cows can be successfully trained to remain within designated VF inclusion areas and avoid VF exclusion feeding zones; 2) animals receiving a greater number of VF cues will require prolonged exposure to adapt properly to VF schedules; 3) animals are capable of shifting to alternative locations when access to preferred feeding areas are temporarily excluded by VF; and 4) once VF exclusions are removed, AH and RC cows quickly revisit previously excluded areas, reflecting a transient effect of VF cues. The specific objectives were to compare breed-level differences in mobility, distribution, and behavior responses as a function of the number of auditory warnings and stimulation electric pulses received during the training phase.

## Materials and methods

The study was conducted at the USDA-ARS Jornada Experimental Range in Las Cruces, NM (32°37'N; 106°40'W) from November 21 to December 9, 2023. Protocols for animal handling, use, and training were reviewed and approved by the New Mexico State University’s Institutional Animal Care and Use Committee (protocol # 2301000148) on March 3, 2023.

### Animals

The animals used in this study included desert-adapted AH crossbred cows and RC cows born and raised at the Jornada ranch. Thirty AH (442 ± 93.1 kg) and 30 RC (365.5 ± 65.8 kg) non-lactating cows, aged 4 to 10 yr and naïve to the VF system, were randomly selected for the study and collared with Nofence collars (Batnfjordsøra, Norway) on October 7 and October 18, 2023, respectively. The neck strap and chains were adjusted to allow a 5 cm gap between the collar and the cow’s neck. Cows were kept in pastures adjacent to headquarters with the devices deactivated until the study began (44 and 33 d for AH and RC, respectively) to allow animals to become accustomed to wearing collars.

### Devices

Nofence C2.2 VF collars (https://www.nofence.no/en-us/) were used in this study. The devices consist of a durable plastic weather resistant enclosure containing a circuit board with microcontrollers, a GNSS receiver, a tri-axial accelerometer, and a buzzer and electric pulse module. The collar is powered by two micro solar panels and a rechargeable 3.6 V lithium-ion battery (20.6 Ah). Adjustable metal chains on each side of the neck are used to deliver electric pulses. The total weight of the unit is approximately 1,446 g. The speaker module is programmed to deliver a rising auditory warning signal to a maximum of 82 dB. The GNSS receiver communicates using GPS and GLONAS satellite constellations. Wireless communication is implemented using LTE Cat-M1 and 2G protocols and short-range Bluetooth. The collars also have the capacity to store information on board for 2 wk in case LTE communication is interrupted. Bluetooth connectivity allows the operator to remove active fencing configurations when LTE communication is restricted or limited. The collar is controlled using a programmable smartphone application (Nofence App). Through this interface, the user can create virtual pastures, assign or remove collars from pastures, check the power and communication state of collars, track animals in real-time, and monitor the enforcement of VF cues.

The Nofence collars rely on the GNSS module to monitor animal position with reference to a programmable VF polygon. If the animal approaches the virtual boundary, the collar triggers the auditory warning, starting at around 3–5 m from the virtual line. The Nofence collars use two modes: teaching and standard. In teaching mode, the auditory warning ceases as soon as the animal comes to a stop or slightly changes its orientation relative to the virtual boundary. After 20 successful training events, the collar automatically switches to standard operating mode. In standard mode, auditory warnings cease once the animal has retreated approximately 2 m back into the VF containment zone. If the animal trespasses the virtual fence line, an electric pulse of 3 kV lasting 1 sec occurs (Goliński et al. 2022). If three consecutive auditory warnings and electric pulses are ignored, an escape event is reported, and the electric fencing module is automatically turned off. The VF module is restored only if the animal reenters the VF enclosure zone.

The collars gather, log, and report data to a network and data server accessible online through the Nofence desktop App. The reported data include timing and duration of auditory warnings, occurrence of electric pulses and escapes, real-time location at 15-min intervals, and recorded locations and activity at 30-min intervals. The activity is determined by tri-axial accelerometers and reported as a one-dimensional motion index. Additionally, the geographic coordinates of polygons for each virtual pasture are available through the desktop App.

### Training protocol

**Figure 1:**
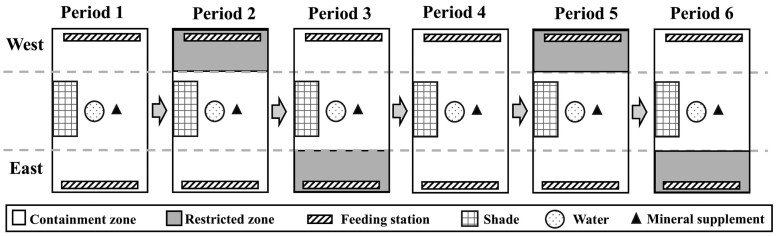
Schematic representation of pen configuration and training sequence.

The VF training lasted 18 days. Cows of each breed were randomly divided into three groups of ten animals (n = 3), and each group was randomly assigned to one of six pens. Initial body weight and body condition score (1–9 scale; determined visually by trained observers) were recorded at study onset. Pens (∼60 x 25 m) were adjacent and constructed of 1.8 m metal panels. Each pen provided access to fresh, clean water and shade located in the center (Figure 1). During the study, cows were fed wheat hay *ad libitum* twice daily in feed stations placed at the east and west ends of the pen. Additionally, free-choice mineral supplements were available near each water trough (Figure 1). Each pen was divided virtually to include a containment zone comprising ∼ ¾ of the area and a restricted zone comprising the remaining ∼¼ of each pen, which alternated between the feeding stations in the east and west ends, respectively (Figure 1). This configuration assured water, mineral supplement, and shade were always available inside the containment zone. When the cows were in this zone, they did not receive auditory warnings or electric pulses. If cows trespassed into the restricted zone, they received the auditory warnings alone or the auditory warnings followed by the electric pulse, respectively. The containment zone had access to an alleyway with a squeeze chute for animal handling and collar fitting.

The training protocol consisted of six consecutive periods (1–6) of three days (1–3) each (Figure 1; Table 1). On day 1 of period 1, the cows were moved to their respective pens. In this period, no virtual boundaries were activated inside the pens. This allowed cows to become familiar with the location of feeding stations, water sources, supplements, shade structures, and presence of personnel. During period 2, the restricted zone was activated at the feeding station located on the west end of pens and switched to the east end during period 3. This switch was performed to test for a residual effect of the VF or association of cueing signals with a specific location within the pen. During period 4, collars were deactivated. This period served as an extinction phase to evaluate persistent effects of VF cueing. During periods 5 and 6, collars were reactivated as in periods 2 and 3, respectively. At the end of day 3 of period 6, all collars were deactivated, final body weight and body condition score of cows were recorded, and changes of body weight and body condition scores during the study were calculated.

**Table 1:** Study timeline.

Study period	Study day	Virtual fencing	Study phase	Management activity
**Initial**	1	Deactivated	Initialization	Body weight and body condition score measurement. Grouping of ­animals and allocation of groups to pens.
**1**	1-3	Deactivated	Adaptation	No virtual fences were activated. No restricted zone is implemented during this phase.
**2**	4-6	Activated	Learning	Virtual fence activated to exclude the west feeding station. Restricted zone on the west end.
**3**	7-9	Activated	Learning	Virtual fence switched to exclude the east feeding station. Restricted zone on the east end.
**4**	10-12	Deactivated	Extinction	No virtual fences were activated. No restricted zone is implemented during this phase.
**5**	13-15	Activated	Testing	Virtual fence activated to exclude the west feeding station. Restricted zone on the west end.
**6**	16–18	Activated	Testing	Virtual fence switched to exclude the east feeding station. Restricted zone on the east end.
**Final**	18	Deactivated	Completion	No virtual fence activated. Body weight and body condition score ­measurement. End of study.

During the study, two GoPro Black Hero 9 (GoPro, San Mateo, CA) video cameras were installed in the northwest and southwest corners of the two adjacent central pens to monitor cattle. These cameras captured the entire area of the pens and were used to monitor and document the daily activity and behavior of cows during daylight hours.

### Data processing

The data recorded by collars were downloaded from the Nofence App in CSV format. For each of the pens, period and period day, response variables were calculated as the daily average per head. These variables included the number of auditory warnings and electric pulses, the ratio of electric pulses to auditory warnings, and the percentage of animal locations within the VF containment zone during periods when the virtual fence was active. In addition, the percentage of animal locations at the central loafing area, the east and west feeding stations, and animal activity were evaluated throughout all periods. The horizontal accuracy of animal locations registered during the study averaged 5.9 m; therefore, a buffer zone of 6 m was used for calculations. Animal locations and coordinates of virtual boundaries were projected to the Universal Transverse Mercator zone 13 N using a WGS 84 datum format. All determinations and calculations of geographic position data were conducted using open-source QGIS 3.36.1 software (https://qgis.org). The daily animal activity was the sum of all 30-min animal activity reports recorded daily for each cow.

### Statistical analysis

Statistical analyses were performed using SAS^®^ Studio software (SAS Institute Inc., Cary, NC, USA). The study was conducted using a completely randomized design with three replications. In this design the experimental unit is the pen of cows, while the observational unit is each individual cow (Bello et al. 2016). Analysis of variance was conducted using repeated measure mixed models using the PROC MIXED procedure (Littell et al. 2006) to evaluate the fixed effects of breed (AH and RC), period (1–6), day nested within period (1–3), and the two-way and three-way interactions. All models were fitted with the Kenward and Roger (1997) method for determination of degrees of freedom of the error term used to test the main fixed effects and their interactions. The study day (1–3), nested within each period (1–6) was treated as the repeated-measures variable, with the pen x breed x period specified as the subject term. The best fit of covariance structure among compound symmetry, first-order autoregressive, unstructured, heterogeneous autoregressive, and heterogeneous compound symmetry models was chosen according to the smaller Akaike’s Information Criteria value. An alpha of 0.05 was used to determine significance of main effects and interactions, whereas an alpha of 0.10 was used to indicate a tendency toward significance. Significant interactions were further analyzed by the slice method (Winer 1971). Means were separated using LSD when main effects were significant (*P* < 0.05) or tendency (*P* < 0.1).

The initial and final body weight and body condition score of AH and RC cows were analyzed using a pairwise T-Test comparisons of breeds (*P *< 0.05).

## Results

### Performance of collars

During the experiment, three collars malfunctioned. The first collar experienced hardware damage that prevented delivering electric pulses. This failure was detected on day 2 of period 3 in a collar assigned to one RC cow. The other two faulty collars developed a firmware issue that resulted in loss of cellular connectivity. One of these collars failed on day 3 of period 5 and the other on day 2 of period 6. Both collar failures occurred on AH cows belonging to ­different pens. Faulty collars were replaced as soon as the problems were detected. However, data from faulty collars with incomplete periods were not used in the final calculations and analyses. A total of 2,646 location points out of the 108,896 location points registered during the study were detected outside the 6-m buffer zone around pens and were therefore discarded from the final analysis.

### Collar cueing variability

One AH cow and six RC cows did not register an electric pulse on day 1 of period 2 when the VF restricted zones were activated for the first time. Except for one RC, all cows received at least one electric pulse in subsequent periods 3, 5, and 6. On day 2 of period 6, there were no electric pulses registered on RC cows, coinciding with the low number of auditory warnings reported for this breed. A maximum of 15 electric pulses were registered on one AH cow on day 1 of period 2. This cow also received 34 auditory warnings on the same day. A maximum of 8 and 6 electric pulses in one day were registered on all other AH and RC cows, respectively. One RC cow received no electric pulses or auditory warnings during the study. On the other hand, one RC cow received 19 auditory warnings without a single electric pulse on day 1 of period 3. Similarly, one AH cow received 14 auditory warnings without registering an electric pulse on day 2 of period 6.

### Collar response variables

Breed x period x day interactions were detected for auditory warnings, electric pulses, and locations inside of the VF containment zone (*P *< 0.05). However, no interactions among main factors (*P *> 0.05) on the ratio of electric pulses to auditory warnings were observed (Table 2).

**Table 2:** Results of the ANOVA for main effects, two-way and three-way interactions among breed, period and day within period for auditory warnings, electric pulses, ratio of electric pulses to auditory warnings and percentage of locations inside the containment zone along with the corresponding degrees of freedom and covariance structure of models.

Variable^1^	Main^2^	df	*P-*value	Two-way	df	*P-*value	Three-way	df	*P-*value	Cov^3^
**AW**	B	1	0.011	B x P	3	0.114	B x P x D(P)	6	0.010	ARH
	P	5	0.006	B x D(P)	2	0.424				
	D(P)	2	0.000	P x D(P)	6	0.001				
**EP**	B	1	0.000	B x P	3	0.003	B x P x D(P)	6	0.011	ARH
	P	5	0.000	B x D(P)	2	0.024				
	D(P)	2	0.000	P x D(P)	6	0.000				
**RT**	B	1	0.022	B x P	3	0.671	B x P x D(P)	6	0.845	ARH
	P	5	0.000	B x D(P)	2	0.285				
	D(P)	2	0.000	P x D(P)	6	0.102				
**IN**	B	1	0.015	B x P	3	0.075	B x P x D(P)	6	0.015	CSH
	P	5	0.018	B x D(P)	2	0.409				
	D(P)	2	0.000	P x D(P)	6	0.000				

1 AW: Auditory warnings; EP: Electric pulses; RT: Ratio EP/AW; IN: Percentage of location inside the containment zone

2 B: Breed; P: Period; D(P): Period Day nested within period

3 Cov: Covariance structure; ARH: Heterogeneous autoregressive; CSH: Heterogeneous compound symmetry.

### Auditory warnings

**Figure 2:**
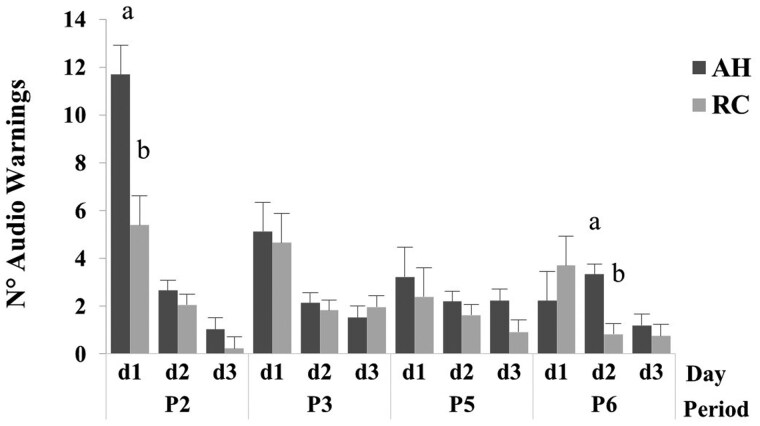
Mean and SE of auditory warnings for Angus-Hereford (AH) and Rarámuri Criollo (RC) cows by period (P2, P3, P5, and P6) and day (d1–d3). The virtual fence was activated to exclude feeding stations on the west (P2 and P5) and east (P3 and P6) side of the pens, respectively. Columns with different letters indicate differences between breeds for the period and day combination LSD (*P *< 0.05).

A breed x period x day interaction (*P *< 0.05) was detected for the auditory warnings (Table 2). The number of auditory warnings differed (*P *< 0.05) between breeds (Figure 2; Table S1). The AH cows had a greater number of auditory warnings than RC on day 1 of period 2 and day 2 of period 6. Within breed, the number of auditory warnings was greater (*P *< 0.05) for AH cows on day 1 of period 2 than day 1 of period 3, period 5, or period 6, respectively. The number of auditory warnings for AH cows was greater (*P *< 0.05) on day 1 than day 2, and on day 1 and 2 than day 3 in period 2, day 1 than day 2 or day 3 in period 3, and day 2 than day 1 or day 3 in period 6, respectively. The number of auditory warnings for RC cows was greater (*P *< 0.05) on day 1 than day 2, and on day 1 and 2 than day 3 in period 2, respectively. The number of auditory warnings declined (*P *< 0.05) across the period when VF was activated (periods 2, 3, 5 and 6) and after switching the restricted zone from the west (periods 2 and 5) to the east feeding station (periods 3 and 6).

### Electric pulses

**Figure 3:**
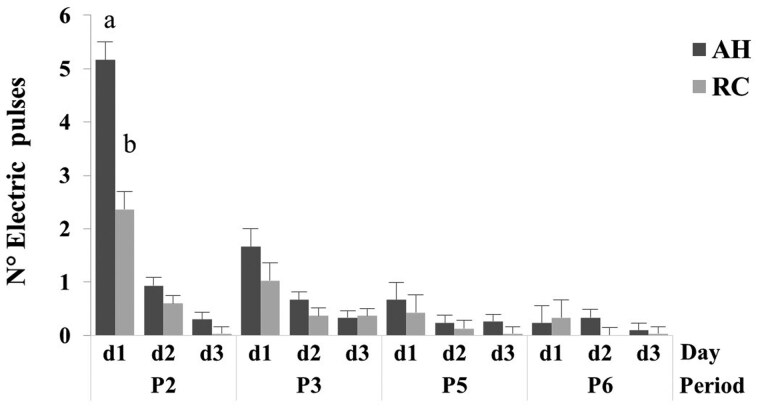
Mean and SE of electric pulses for Angus-Hereford (AH) and Rarámuri Criollo (RC) cows by period (P2, P3, P5, and P6) and day (d1-d3). The virtual fence was activated to exclude feeding stations on the west (P2 and P5) and east (P3 and P6) side of the pens, respectively. Columns within periods and period day followed by a different letter indicate differences between breeds (LSD; *P *< 0.05).

The number of electric pulses was affected by breed x period x day interaction (*P *< 0.05; Table 2). The number of electric pulses was greater (*P *< 0.05) for AH than RC cows on day 1 of period 2. Within breed, the number of electric pulses registered by AH was greater (*P *< 0.05) on day 1 of period 2 than period 3, day 1 of period 2 and period 3 than period 5 and period 6, and on day 2 of period 2 and period 3 than period 5 and period 6, respectively. The electric pulses registered by RC were greater (*P *< 0.05) on day 1 of period 2 than period 3, period 5, or period 6, respectively. Likewise, the electric pulses registered by AH were greater (*P *< 0.05) on day 1 than day 2 and day 3 in period 2, and day 1 than day 2 or day 3 in period 3, respectively (Figure 3). The number of electric pulses for RC cows were greater (*P *< 0.05) on day 1 than day 2 or day 3 of period 2, respectively (Figure 3). As with auditory warnings, the number of electric pulses decreased across periods and test days within periods for both breeds (Figure 3; Table S2).

### Ratio of electric pulses to auditory warnings

**Figure 4:**
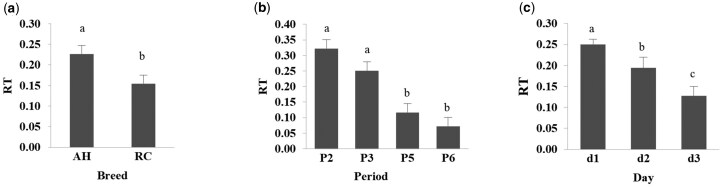
Mean and SE of the ratio (RT) of electric pulses to auditory warnings for: (a) Angus-Hereford (AH) and Rarámuri Criollo (RC) cows, (b) training periods (P2, P3, P5, and P6) and (c) training period day (d1–d3) with virtual fences activated to exclude feeding stations in the west (P2 and P5) or east (P3 and P6) side of the pens. Vertical bars for the breed, period and day main effects followed by a different letter indicate significant differences (LSD; *P *< 0.05).

There were no interactions (*P *> 0.05) among breeds, periods, or days for the ratio of electric pulses to auditory warnings (Table 2). The ratio of electric pulses to auditory warnings differed (*P *< 0.05) between breeds, with AH cows having a greater value than RC cows, respectively (Figure 4a; Table S3). The ratio of electric pulses to auditory warnings also differed (*P *< 0.05) among training periods with greater values in periods 2 and 3 than periods 5 and 6, respectively (Figure 4b; Table S3). The ratio of electric pulses to auditory warnings also differed (*P *< 0.05) among training days, with day 1 greater than day 2, and day 1 and 2 greater than day 3 (Figure 4c; Table S3).

### Virtual fencing containment

**Figure 5:**
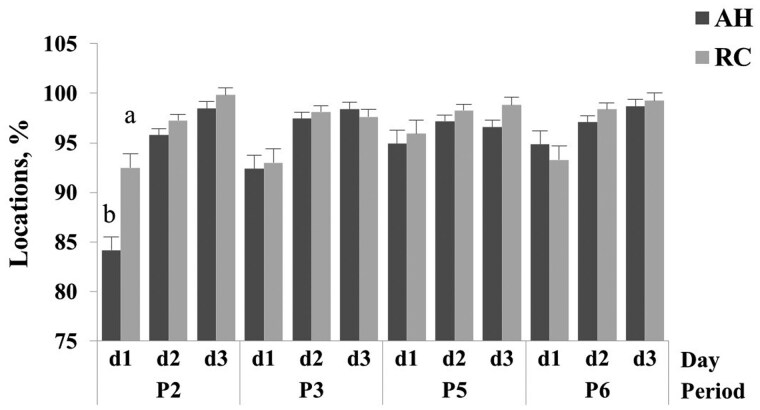
Mean and SE of percentage of animal locations inside the containment zone for Angus-Hereford (AH) and Rarámuri Criollo (RC) cows by period (P2, P3, P5, and P6) and period day (d1–d3). The virtual fence was activated to exclude feeding stations on the west (P2 and P5) and east (P3 and P6) side of the pens, respectively. Columns within periods and period days followed by a different letter indicate differences between breeds (LSD; *P *< 0.05).

The breed x period x day interaction was significant (*P *< 0.05). Containment by the VF was greater (*P *< 0.05) for RC than AH on day 1 of period 2, respectively. No differences were observed (*P *> 0.05) between breeds for any other training period by day combination (Figure 5; Table S4). Containment by the VF was lower (*P *< 0.05) for AH on day 1 of period 2 than day 1 of period 3, period 5, or period 6, respectively (Figure 5; Table S4). The containment by VF differed (*P *< 0.05) among training days, being lower (*P *< 0.05) for AH on day 1 than day 2 or day 3 in period 2 and day 1 than day 2 or day 3 in period 3, and day 1 than day 3 in period 6, respectively. The containment of RC cows also differed (*P *< 0.05) among training days, being lower (*P *< 0.05) on day 1 than day 2 or day 3 in period 2, day 1 than day 2 or day 3 in period 3 and day 1 than day 2 or day 3 in period 6, respectively (Figure 5; Table S4).

### Animal response variables

There was a tendency (*P *< 0.10) for a breed x period x day interaction for the distribution of animal locations in the east and west sides. A significant breed x period x day interaction (*P *< 0.05) was found for the proportion of animal locations registered in the central inclusion zone of the pens and for the animal activity registered by the motion index (Table 3).

**Table 3:** Results of the ANOVA for main effects, two-way and three-way interactions among breed, period and day within period for the proportion of animal locations registered in the east and west feeding stations and central loafing area of training pens, and motion index.

Variable	Main^1^	df	*P*-value	Two-way	df	*P*-value	Three-way	df	*P*-value	Cov^2^
**Proportion of animal locations, %**										
**East feeding station**	B	1	0.012	B x P	5	0.076	B x P x D(P)	10	0.076	UN
	P	5	0.000	B x D(P)	2	0.013				
	D(P)	2	0.000	P x D(P)	10	0.000				
**Central loafing area**	B	1	0.117	B x P	5	0.904	B x P x D(P)	10	0.045	CS
	P	5	0.000	B x D(P)	2	0.182				
	D(P)	2	0.000	P x D(P)	10	0.008				
**West feeding station**	B	1	0.615	B x P	5	0.156	B x P x D(P)	10	0.057	AR
	P	5	0.000	B x D(P)	2	0.261				
	D(P)	2	0.408	P x D(P)	10	0.000				
**Animal Activity**										
**Motion index**	B	1	0.000	B x P	5	0.789	B x P x D(P)	10	0.000	CS
	P	5	0.070	B x D(P)	2	0.279				
	D(P)	2	0.000	P x D(P)	10	0.009				

1 B: Breed; P: Period; D(P): Period Day nested within period

2 Cov: Covariance structure; CS: Compound symmetry; UN: Unstructured; AR: Autoregressive.

### Animal location

**Figure 6:**
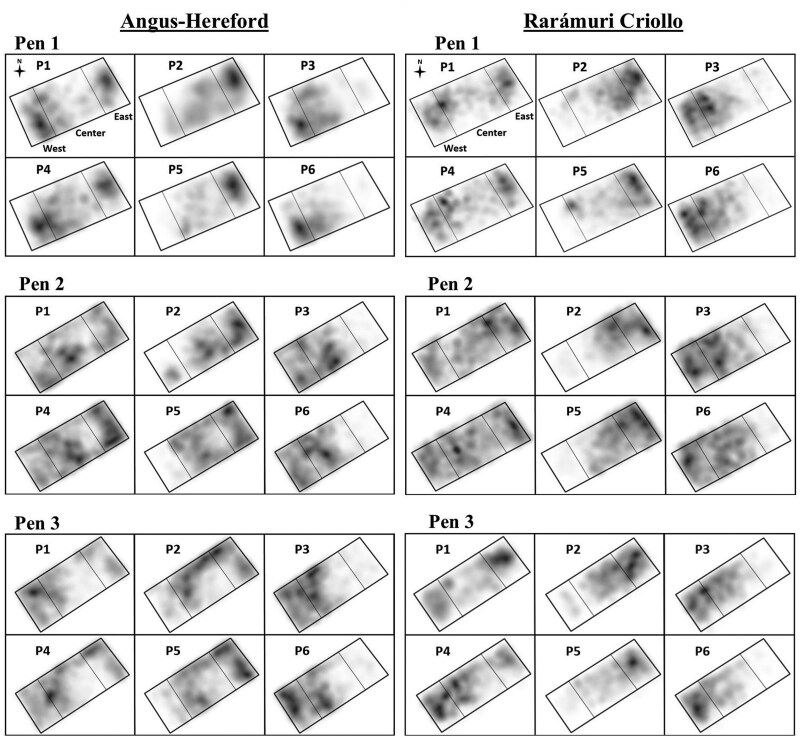
Distribution in virtual fence training pens (Pen 1–3) of Angus-Hereford (AH) and Rarámuri Criollo (RC) cows across six training periods (P1–P6). The virtual fence was deactivated (P1 and P4) or activated to exclude feeding stations on the west (P2 and P5) and east (P3 and 6) side of the pens, respectively.

**Figure 7:**
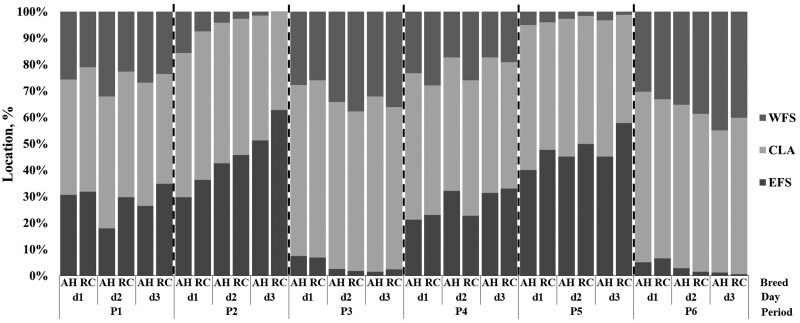
Percentage of animal locations by period (P1–P6) and period day (d1–d3) of Angus-Hereford (AH) and Rarámuri Criollo (RC) cows either in east (EFS) and west (WFS) feeding stations or central loafing area (CLA) of virtual fence training pens. The virtual fence was either inactive (P1 and P4) or activated to exclude feeding stations on the west (P2 and P5) or east (P3 and P6) side of the pens, respectively.

The distribution of animals inside pens was affected by the experimental design (Figure 6). Therefore, differences in the distribution of animal locations between breeds were analyzed within training periods and days. The presence of the cows at feeding stations was influenced by the VF containment protocol (Figures 6 and 7; Table S5). When VF was inactive, cows spent 27.9% and 23.6% of their time at the east and west feeding stations, respectively (Figure 7). However, during the activation of VF on the west side in periods 2 and 5 and east side in periods 3 and 6, the time spent at the excluded feeding stations decreased to 4.18% and 3.45%, and the time spent at the alternative feeding station increased by 46.2% and 32.1% on average, respectively (Figure 7).

There was a tendency (*P *< 0.1; Table 3) for a breed x period x day interaction on the proportion of locations at the east and west feeding stations. The proportion of animal locations in the east feeding station area was greater (*P *< 0.1) for RC than AH on day 2 and day 3 of period 1, day 1 and day 3 of period 2, and day 1 and day 3 of period 5, respectively (Figure 7; Table S5). The proportion of animal locations in the east feeding station area was greater (*P *< 0.1) for AH than RC cows on day 2 of period 4 (Figure 7; Table S5). Conversely, the proportion of animal locations in the west feeding station area was greater (*P *< 0.1) for AH than RC cows on day 2 of period 1 and day 1 of period 2 respectively (Figure 7; Table S5). In contrast, the proportion of animal locations on the west feeding station area was greater (*P *< 0.05) for RC cows than AH cows on day 2 of period 4 (Figure 7; Table S5). The proportion of animal locations in the central loafing area of pens was affected (*P *< 0.05) by a breed x period x day interaction (Table 3), with AH cows spending a greater (*P *< 0.05) proportion of time than RC in the central loafing area on day 3 of period 2, respectively (Figure 7; Table S5).

### Animal activity

**Figure 8:**
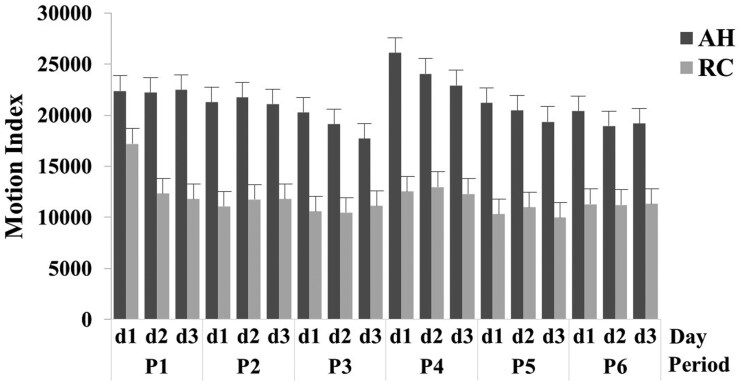
Mean and SE of animal activity of Angus-Hereford (AH) and Rarámuri Criollo (RC) measured by the motion index by period (P1–P6) and day (d1–d3). The virtual fence was either inactive (P1 and P4) or activated to exclude feeding stations on the west (P2 and P5) or east (P3 and P6) side of the pens, respectively.

There was a breed x period x day interaction (*P *< 0.05) for the animal activity (Table 3). The motion index indicated greater (*P *< 0.05) animal activity for AH than RC across all periods and days (Figure 8; Table S6). The RC cows had greater (*P *< 0.05) animal activity in day 1 of period 1 than day 1 of period 2, 3, 4, 5, or 6, respectively. The animal activity of AH cows was greater (*P *< 0.05) on day 1 than day 3 of period 3 and day 1 than day 3 of period 4, respectively. The RC cows had greater (*P *< 0.05) animal activity on day 1 than day 2 or day 3 of period 1, respectively (Figure 8; Table S6).

### Body weight and body condition score

No differences (*P *> 0.05) were found between initial and final body weight or body condition score of AH cows (Table S7). The initial and final body weight of RC cows did not differ (*P* > 0.05), but the final body condition score (5.7) was greater (*P *< 0.05) than the initial body condition score (5.3) for the RC cows (Table S7).

## Discussion

This study evaluated the training and adaptation of AH and RC cows to a dynamic implementation of VF schedules during a controlled feed challenge. Results supported the four hypotheses tested. Both AH and RC cows successfully learned to respect the virtual boundaries and remain within designated VF inclusion areas while avoiding feeding on alternative feeding stations excluded by VF. Animals that did initially receive a greater number of VF cues did require prolonged exposure to properly respect VF limits by minimizing numbers of electric pulses received relative to the number of auditory warnings. Likewise, cows were most capable of shifting to alternative locations when access to preferred feeding areas were temporarily excluded by VF. Finally, once a previous VF exclusion were removed, both AH and RC cows quickly revisited previously excluded areas, indicating plastic behavioral adaptations to VF schedules and reflecting transient effects of VF cues on cows.

Proper animal training is crucial for applying VF systems successfully (Anderson 2007; Umstatter 2011; Colusso et al. 2020). Consistent with previous behavioral ecology theory (Koolhaas et al. 1999), the results support the hypothesis that mobile and more passive responder AH cows (ie, requiring greater VF cueing for containment) were more dynamic and interacted with the virtual boundaries more frequently, receiving a greater number of auditory warnings and electric pulses than RC cows upon the first exposure to VF. The results also support the hypothesis that as the study progressed, both active responder RC cows (ie, requiring less VF cues) and passive responders (AH) properly learned and adapted to shifts in VF configuration. The consistently low ratio of electric pulses to auditory warnings by the completion of the study indicates that both AH and RC cows properly learned and adapted to the containment enforced by the Nofence collars, although they did so by using different strategies.

In this study, cows were challenged with changing rather than permanent boundaries in an effort to simulate dynamic VF configurations encountered in real-world applications. The present study confirms that providing sufficient exposure and cueing pressure to all animals is essential for them to learn the association between the auditory warning and conditioning electric pulse (Lee et al. 2009; Colusso et al. 2020; Confessore et al. 2022).

Once cows became familiar with the training and standard operating mode of Nofence collars, they still required receiving standard auditory warnings followed by electric pulse cycles to reinforce learning associations (Laca 2009). Such reinforced learning may enable cows to quickly adapt to environmental changes (eg, vegetation shifts) and to virtual boundary modifications. First, the animals rapidly learned to avoid areas newly enclosed by the VF (period 1 → period 2). Second, experienced animals adapted quickly when the VF boundaries shifted between the west and east feeding locations (period 2 → period 3 and period 5 → period 6). Similarly, they promptly revisited feeding areas once VF restrictions were removed and avoided previously accessible feeding locations once new VF enclosures were applied (period 2 → period 3 and period 5 → period 6). Finally, animals quickly returned to previously excluded areas during the extinction phase (period 4). Remarkably, this revisit occurred regardless of the order or length of prior VF exclusion. These findings suggest that livestock responses to VF are adaptive, offering a flexible approach for dynamically including or excluding animals from preferred zones. The results are consistent with those of previous conditioning trials in which conditioned ewes and lambs (Black-Rubio et al. 2007) rapidly avoided locations paired with an electric shock but quickly revisited previous shock-cued areas upon shock signal extinction, thereby supporting adaptive learning.

Regular reinforcement with VF cues appears to be important for the maintenance of location avoidance behaviors (Laca 2009). However, the effectiveness of VF reinforcement and the animal’s ability to adapt to changing virtual pasture configurations can be influenced by multiple factors, including pasture size, terrain, vegetation cover, physical fences or natural barriers, water points, shade, forage depletion, animal physiological state, and collar issues (Boyd et al. 2023; Ochoa et al. 2024; Fuchs et al. 2025; Silber et al. 2025).

### Collar performance

Of the 60 collars deployed, three collars (5%) malfunctioned, one due to hardware failure and two due to firmware issues. A video review indicated the hardware malfunction involved a loose mounting bracket and electrodes, allowing the collar to issue auditory warnings but not delivering electric pulses. Consequently, the cow was not effectively excluded from the restricted zone, and 35 false electric pulses were reported on the first day. The cow showed no sign of injury or distress due to collar failure. This incident suggests that naïve animals cannot reliably respond to auditory warnings alone. Rather, proper associative learning requires experiencing both auditory warning cues alone and auditory warnings preceding an electric pulse (Launchbaugh and Howery 2005; Lee et al. 2009; Confessore et al. 2022). No other animals followed the affected cow, indicating that all other cows responded properly to the enforced VF containment. For the other two failed collars, firmware failures were noted. Unexpected alerts indicating an “unknown” operational state were received. These devices remained offline and had a faulty battery controller reading 0 V. These collars were not able to update location data or receive new VF configurations via GSM or Bluetooth. In this state, it was not possible to modify the collar configurations or confirm whether the containment functions were active. Consequently, the faulty collars were replaced immediately.

### Collar cueing variability

Differences in activity and associated responses to VF were observed between breeds and among individuals. These differences were most evident on the first day of VF exposure (day 1 of period 2). More active responders (ie, RC cows)—those receiving or needing fewer cues to respond—may have been influenced by spatial displacement, social interactions with peers (Keshavarzi et al. 2020), or low social rank (Coussi-Korbel and Fragaszy 1995). Similarly, one or a few cued individuals may induce fear or caution in nearby animals (Keshavarzi et al. 2020), indirectly reinforcing avoidance of VF-enclosed areas. Eventually, such social learning avoidance can lead to heightened vigilance, reduced movement (Laundré et al. 2010), and low frequency of interactions with VF boundaries (Campa Madrid et al. 2025). However, other shock-conditioning studies conducted with beef steers indicate that social learning of location avoidance is not very strong among peers (Cibils et al. 2008).

Despite temporal differences, all animals (but one RC cow) eventually experienced the sequence of auditory warning and electric pulse, although the timing and frequency of cues varied. This variation may be associated with a different risk awareness, preference, and willingness to explore feeding stations (ie, eating drive), or social contexts. The fact that some animals did not interact with the boundaries could be driven by the social hierarchy among individuals, which was more notorious among RC cows. Under this concept, follower individuals could copy and mimic reactions of leader animals, reducing the need to personally experience VF cues (Coussi-Korbel and Fragaszy 1995). Keshavarzi et al. (2020) concluded that responses to VF can be socially facilitated through observation of peers that may not necessarily rank as leaders. In this experiment, social learning may have occurred when animals mimicked the experiences or behaviors of peers within the same pen or in adjacent pens (Sato 1982; Nogues et al. 2024). Anecdotally, it was observed that animals of one pen turned occasionally into the inclusion zone upon hearing collar auditory warnings coming from peers in the same pen or from adjacent pens. Social dynamics, including leader-follower interactions, could influence individual responses to VF and this could be the subject for future VF manipulation studies. The inherent uniqueness of each trained animal, due to its individual risk assessment and behavioral tendency (Provenza et al. 2003; Bushby et al. 2018), likely underlies these responses. High animal-to-animal variability to VF cues has also been reported in other training studies using animal groups or individuals (Lomax et al. 2019; Keshavarzi et al. 2020; Aaser et al. 2022). For example, Nyamuryekung’e et al. (2023) and Campa Madrid et al. (2025) reported effective VF containment even for in-training animals that received cues ranging from zero to 10.

### Collar response variables

The number of auditory warnings was affected by an interaction between breeds, periods, and days, with AH receiving more auditory warnings at the beginning of training than RC. This observation supports the hypothesis that factors, such as activity, temperament, feeding behavior, and risk assessment, differ between breeds. In addition, AH continued to experience more auditory warnings in the last period of the study.

Despite differences in auditory warnings, the number of electric pulses did not differ between breeds toward the end of the study. This result may indicate that both AH and RC cows learned to respond to the collar auditory warnings while avoiding electric pulses, but through strategies involving a different degree of exposure to VF and cue reinforcement. Consequently, vigilant, active responders (RC) required less exposure to VF boundaries to learn to avoid electric pulses, whereas bolder, passive responders (AH) required greater exposure to reinforcement to learn how to rely on auditory warnings and avoid electric pulses (Koolhaas et al. 1999; Welp et al. 2004; Aaser et al. 2022). These findings support the hypothesis that all animals learn how to properly react to auditory warnings to avoid electric pulses and that animals with different reactiveness may have distinct mechanisms to cope with negative reinforcement.

The results also showed a rapid decrease in the number of auditory warnings from day 1 to day 3 of period 2 compared to other periods and period days, suggesting that the associative learning of VF is a relatively rapid process. Throughout the study, all animals (but one) interacted with the VF boundaries, with the number of electric pulses per head declining rapidly over time. These results align with the findings of Nyamuryekung’e et al. (2023) and Campa Madrid et al. (2025), who reported decreasing auditory warnings from Nofence collars after the initial interaction with a new VF boundary in a training pen or pasture, respectively. Lee and Campbell (2021) also described the conditioning learning of VF as a rapid process. In addition, continuous interaction with boundaries is highly desirable for reinforcement learning (Laca 2009). A greater number of auditory warnings is not necessarily an undesirable result if the number of electric pulses is minimal. A low ratio of electric pulses to auditory warnings may indicate that the associative learning of VF cues was successful. The total number of auditory warnings refers to the frequency of interaction with boundaries, while other indicators, such as the number of electric pulses, the ratio of electric pulses to auditory warnings, the changes in animal activity, or alterations of foraging behavior may be required to further evaluate VF learning success.

The number of electric pulses decreased as the duration of training progressed, which is aligned with findings reported in previous studies with cattle trained in pens (Nyamuryekung’e et al. 2023) and in small paddocks (Confessore et al. 2022; Hamidi et al. 2024; Fuchs et al. 2025). However, low numbers or absence of electric pulses alone do not necessarily indicate adequate learning. For example, a low number of electric pulses could reflect low VF pressure (ie, animals in large virtual enclosures) or location avoidance behaviors. In this study, both breeds received the greatest number of electric pulses when previously unknown VF locations were activated, even though breeds differed. Although AH received a greater number of auditory warnings than RC cows, both had the same low number of electric pulses by study end.

In VF, success can be defined by the number of auditory warnings not associated with the delivery of electric pulses (Eftang et al. 2022). Likewise, odds of VF success can be calculated by ratios or indices that assess relationships between electric pulses received and auditory warnings produced. For example, a ratio of electric pulses to auditory warnings below 0.5 will indicate increased response to auditory warnings and less reliance on electric pulses. However, while extremely useful, this ratio will not fully account for the absolute values of auditory warnings and total electric pulses received. Therefore, the overall intensity of interactions with virtual boundaries may not be fully determined using success ratios (Eftang et al. 2022, 2023). Although there was no interaction of breed with time for the ratio of electric pulses to auditory warnings, the AH cows had both greater number of auditory warnings and electric pulses and therefore a higher ratio of electric pulses to auditory warnings than RC, indicating passive responder AH cows registered greater exposure and required greater reinforcement through time. This finding supports the hypothesis that calm RC cows had different temperaments and level of risk assessment than AH cows. The decline of the ratio of electric pulses to auditory warning across period days (day 1 → day 3) and study periods (period 2 → period 6) supports progressive learning by the increased reliance on auditory warnings vs. the required number of electric pulses reinforcement. These results are aligned with findings reported by Nyamuryekung’e et al. (2023), who found declining relative numbers of electric pulses through time. The results also support that the adaptation of animals to VF can be rapid and is best described as an adaptive self-learning process (Lee and Campbell 2021).

### Containment of animals

Overall, animal containment was effective when the virtual boundaries were active. On day 1, AH cow containment was 84.2%, and for all subsequent days and periods, containment of both breeds was > 90%. These results support the hypothesis that cows exposed to a feeding challenge (ie, excluding feeding areas) readily adapt to VF exclusion. In addition, the results reinforce the hypothesis that bolder passive responders interact more frequently and need more exposure to VF to learn how the cueing system works (Welp et al. 2004; Aaser et al. 2022). The RC cows were less active and despite being exposed to a feed challenge, their containment was never below 90%. The observed responses to altered VF protocols showed that animals need time to adjust their locations regardless of the location of the excluded zone. Increased containment from day 1 to day 3 suggests that once animals encounter a newly restricted zone, they quickly alter their behavior to search for alternative feed resources (ie, forage at alternative feeding stations), and this foraging plasticity appears to be faster if animals have prior information about the available feeding locations. These results agree with Nyamuryekung’e et al. (2023), who found that cattle spent incrementally greater time at feeding stations established inside a VF containment zone, illustrating the role of temporal reinforcement in progressive learning about VF. Although this training was conducted in relatively small, fenced pens, the feed offered inside VF excluded zones served as an attractant to challenge animals to trespass the virtual boundary. Despite the presence of feed, the VF collar effectively contained animals inside the containment area.

### Animal response variables

The animals altered the distribution inside pens mostly in response to VF schedules, though differences were noted between breeds. As anticipated, the location and temporal schedule of the VF affected cow location. In the absence of active virtual boundaries (period 1 and period 4), the animals distributed evenly throughout the entire pen area, but slight differences were observed between RC and AH cows. The RC cows chose areas of the pen further away from working facilities located west of the training pens. Conversely, AH cows were more willing to distribute evenly, even in proximity to enforced VF feeding sites. On the other hand, both breeds showed almost identical presence in the central loafing area of the pens. During periods where VF was enforced in the east (period 2 and period 5) or west (period 3 and period 6) feeding locations, the RC cows spent most of the time at the end opposite of the VF-excluded area, whereas AH cows remained closer to the exclusion areas enforced by VF. The different distribution pattern may reflect increased fear of VF and more cautious behavior of the RC cows compared to AH cows (Laundré et al. 2010). In fact, RC cows had a lower activity possibly related to their dissimilar behavioral pattern (Cibils et al. 2023), genetic structure and background (Spetter et al. 2025), and/or prolonged natural selection under harsh conditions (Estell 2021). The more active AH cows appeared bolder, remaining closer to the enforced VF areas. These findings support the hypothesis that animals may utilize feed, water, shade, and supplement resources differently in response to VF. During the extinction phase (period 4), the AH cows registered a greater proportion of locations in feeding areas that were previously excluded by VF in period 2 and period 3, which may indicate a faster response to the VF extinction or willingness to explore feeding areas regardless of previous VF schedules. This observation partially supports the hypothesis that apparently bolder AH cows were faster to revisit feeding areas previously excluded by VF. However, the response to shifting VF schedules was incremental for both breeds. The number of locations inside feeding areas excluded by VF decreased across days and periods, whereas the number of locations in the unfenced alternative feeding stations increased across days and periods, supporting progressive learning and avoidance (Confessore et al. 2022; Launchbaugh and Howery 2005).

### Animal activity

Animal motion sensed by tri-axial accelerometers serves as an indicator of the overall animal activity and is extremely sensitive to differences in the time budget al.ocated to feeding, resting, or walking activities (Perea et al. 2025). Behaviors indicating higher inertial movement, such as walking and feeding, represent elevated levels of motion, whereas resting while lying down or standing still or remaining vigilant imply lower inertial movement. Because of the size of the training pens (∼0.15 ha), minimal walking was expected. Consequently, food search and feeding may have represented most of the variation in animal activity reported in this study. Heavier AH cows may have spent more time eating and less time stationary due to their greater nutrient requirements and needs for greater feed intake (NRC 2016), which could partially explain the greater activity of AH cows compared to RC cows throughout the study. Different temperaments among breeds may also partially explain why RC cows were less active and more vigilant than AH cattle (Welp et al. 2004). Likewise, confining cattle to an unfamiliar pen may have reduced movements and increased the vigilance to a greater extent in RC than AH. Roacho Estrada et al. (2023) also found that AH cows spent more time grazing than RC cows while grazing the same desert rangeland, suggesting RC cows may allocate time to other less energetically expensive behaviors. Interestingly, the RC cows showed greater activity on day 1 of period 1, suggesting greater exploration of an unfamiliar area, but a lower exploration as they became familiar with the environment (Welp et al. 2004).

No differences in animal activity were observed when VF were active vs. inactive, suggesting the use of Nofence collars did not adversely alter the regular time budget of animal activities. This result differs with Nyamuryekung’e et al. (2023), who in short-duration VF trials found that Brangus cows reduced their overall activity when virtual enclosures were activated. This discrepancy may relate to different temperaments and responses to VF among breeds, duration of training periods, layout of training pens, physiological stage (lactating vs nonlactating stage), and/or time of year. The AH cows had a numerically greater activity on the first day of period 4, which may indicate greater exploration to cope with changes in VF configuration.

### Body weight and body condition score

Animal welfare is a primary concern with VF applications (Campbell et al. 2019; Lee and Campbell 2021; Hamidi et al. 2022) to the extent that due to current legislation, VF is not allowed as a management practice in some European countries (Aaser et al. 2022; Versluijs et al. 2024). To this end, most studies have evaluated the effects of VF on indicators of animal welfare, using behavioral, physiological, or performance metrics (Campbell et al. 2019; Lee and Campbell 2021; Confessore et al. 2022). For this study, body weight and body condition score were assessed. No change in body weight was noted for either animal breed, suggesting that the present method used to train animals to Nofence collars had minimal effect on animal welfare. Although not statistically significant, numerical gains in body weight were observed for AH, which may reflect differences in the gut fill due to changing diets during the study. A similar initial and final body condition score was observed for AH, while RC cows improved slightly their body condition score by the end of the study (increase of 0.4). This could be due to the subjective nature of body condition score assessments or due to real differences in body tissue deposition associated with changing diets. Longer periods may be required to accurately assess changes in body weight and body condition score more effectively, but the lack of effect on both metrics collectively suggests that the present training protocols did not negatively influence cows. This observation agrees with Campbell et al. (2019) and Hamidi et al. (2022) who reported no adverse effects of VF in animal welfare.

## Conclusion

The training period is a critical phase for the effective implementation of VF on cattle herds. During this time naïve animals must learn to rely on emitted auditory warnings to avoid entering excluded locations enforced by electric pulses. The AH and RC cows responded differently to VF cueing, mainly during the first days of training. These differences were likely related to dissimilar behaviors, animal activity, level of risk assessment, nutritional requirements, and temperament between breeds. These factors may relate to the dissimilar selection history and genetic ancestry between the tested breeds. Despite the initial training differences between breeds, all animals successfully adapted to VF by the end of the study. Progressively greater reliance on collar auditory warnings and lower electric pulses suggested that animals learned to avoid areas enforced by VF, while readily adapting to VF schedules that excluded cattle from previously preferred feed locations.

## Supplementary Material

skag024_Supplementary_Data

## Abbreviations:

VF: Virtual Fencing;
GNSS: Global Navigation Satellite System;
RC: Rarámuri Criollo;
AH: Angus-Hereford
