# Supplementary material for: Differential response of Angus-Hereford and Rarámuri Criollo cattle to a dynamic feeding challenge during the training to an autonomous virtual fencing collar
Source: J Anim Sci. 2026 Feb 3;104:skag024. doi: 10.1093/jas/skag024 (PMC12948931; doi:10.1093/jas/skag024)
Supplement: skag024_Supplementary_Data [file skag024_supplementary_data.zip › 13-Feb-2026_091147_Supplementary_material.docx]

Supplementary material

Table S1. Mean and standard error of Auditory warnings by cow, according to breed, period and day nested within period.

| Period/day^1^ | Angus-Hereford | Rarámuri Criollo | SE |
| --- | --- | --- | --- |
| P 2 |  |  |  |
| d1 | 11.70 | 5.40 | 1.22 |
| d2 | 2.67 | 2.07 | 0.43 |
| d3 | 1.03 | 0.26 | 0.48 |
| P 3 |  |  |  |
| d1 | 5.13 | 4.65 | 1.22 |
| d2 | 2.13 | 1.83 | 0.43 |
| d3 | 1.53 | 1.97 | 0.48 |
| P 5 |  |  |  |
| d1 | 3.22 | 2.40 | 1.22 |
| d2 | 2.18 | 1.63 | 0.43 |
| d3 | 2.25 | 0.93 | 0.48 |
| P 6 |  |  |  |
| d1 | 2.24 | 3.70 | 1.22 |
| d2 | 3.33 | 0.83 | 0.43 |
| d3 | 1.20 | 0.77 | 0.48 |

^1^ P: Period; d: day

Table S2. Mean and standard error of electric pulses by cow, according to breed, period and day nested within period.

| Period/day^1^ | Angus-Hereford | Rarámuri Criollo | SE |
| --- | --- | --- | --- |
| P 2 |  |  |  |
| d1 | 5.17 | 2.36 | 0.33 |
| d2 | 0.93 | 0.59 | 0.15 |
| d3 | 0.30 | 0.04 | 0.13 |
| P 3 |  |  |  |
| d1 | 1.67 | 1.03 | 0.33 |
| d2 | 0.77 | 0.35 | 0.15 |
| d3 | 0.33 | 0.36 | 0.13 |
| P 5 |  |  |  |
| d1 | 0.67 | 0.43 | 0.33 |
| d2 | 0.24 | 0.13 | 0.15 |
| d3 | 0.27 | 0.03 | 0.13 |
| P 6 |  |  |  |
| d1 | 0.24 | 0.33 | 0.33 |
| d2 | 0.33 | 0.00 | 0.15 |
| d3 | 0.10 | 0.03 | 0.13 |

^1^ P: Period; d: day

Table S3. Mean and standard error of the ratio electric pulses/ audio warnings by cow, according to breed, period and day nested within period.

| Period/day^1^ | Angus-Hereford | Rarámuri Criollo | SE |
| --- | --- | --- | --- |
| P 2 |  |  |  |
| d1 | 0.44 | 0.46 | 0.04 |
| d2 | 0.36 | 0.28 | 0.07 |
| d3 | 0.28 | 0.11 | 0.06 |
| P 3 |  |  |  |
| d1 | 0.32 | 0.21 | 0.04 |
| d2 | 0.38 | 0.20 | 0.07 |
| d3 | 0.23 | 0.15 | 0.06 |
| P 5 |  |  |  |
| d1 | 0.18 | 0.19 | 0.04 |
| d2 | 0.11 | 0.08 | 0.07 |
| d3 | 0.09 | 0.05 | 0.06 |
| P 6 |  |  |  |
| d1 | 0.11 | 0.09 | 0.04 |
| d2 | 0.12 | 0.00 | 0.07 |
| d3 | 0.09 | 0.02 | 0.06 |

^1^ P: Period; d: day

Table S4. Mean and standard error of percentage of location registered inside of the inclusion zone by cow, according to breed, period and day nested within period.

| Period/day^1^ | Angus-Hereford | Rarámuri Criollo | SE |
| --- | --- | --- | --- |
| P 2 |  |  |  |
| d1 | 84.17 | 92.50 | 1.36 |
| d2 | 95.80 | 97.27 | 0.62 |
| d3 | 98.47 | 99.83 | 0.73 |
| P 3 |  |  |  |
| d1 | 92.37 | 93.00 | 1.36 |
| d2 | 97.43 | 98.13 | 0.62 |
| d3 | 98.37 | 97.63 | 0.73 |
| P 5 |  |  |  |
| d1 | 94.90 | 95.93 | 1.36 |
| d2 | 97.20 | 98.27 | 0.62 |
| d3 | 96.57 | 98.83 | 0.73 |
| P 6 |  |  |  |
| d1 | 94.83 | 93.3 | 1.36 |
| d2 | 97.07 | 98.40 | 0.62 |
| d3 | 98.67 | 99.27 | 0.73 |

^1^ P: Period; d: day

Table S5. Mean and standard error of percentage of location registered inside of the east and west feeding areas and the center loafing area inclusion area by cow, according to breed, period and day nested within period.

|  |  | East loafing area | | |  | Center loafing area | | |  | West loafing area | | |
| --- | --- | --- | --- | --- | --- | --- | --- | --- | --- | --- | --- | --- |
| Period/day^1^ |  | AH^2^ | RC^3^ | SE |  | AH | RC | SE |  | AH | RC | SE |
| P 1 |  |  |  |  |  |  |  |  |  |  |  |  |
| d1 |  | 30.60 | 31.77 | 2.58 |  | 43.77 | 47.17 | 3.46 |  | 25.67 | 21.07 | 2.58 |
| d2 |  | 17.97 | 29.83 | 2.42 |  | 49.80 | 47.50 | 3.46 |  | 32.23 | 22.67 | 2.58 |
| d3 |  | 26.50 | 34.87 | 3.18 |  | 46.63 | 41.50 | 3.46 |  | 26.93 | 23.60 | 2.58 |
| P 2 |  |  |  |  |  |  |  |  |  |  |  |  |
| d1 |  | 29.80 | 36.33 | 2.58 |  | 54.37 | 56.13 | 3.46 |  | 15.83 | 7.50 | 2.58 |
| d2 |  | 42.57 | 45.83 | 2.42 |  | 53.27 | 51.40 | 3.46 |  | 4.20 | 2.73 | 2.58 |
| d3 |  | 51.20 | 62.73 | 3.18 |  | 47.33 | 37.10 | 3.46 |  | 1.53 | 0.17 | 2.58 |
| P 3 |  |  |  |  |  |  |  |  |  |  |  |  |
| d1 |  | 7.63 | 7.00 | 2.58 |  | 64.63 | 67.00 | 3.46 |  | 27.77 | 25.97 | 2.58 |
| d2 |  | 2.57 | 1.87 | 2.42 |  | 63.10 | 60.30 | 3.46 |  | 34.33 | 37.87 | 2.58 |
| d3 |  | 1.63 | 2.37 | 3.18 |  | 66.20 | 61.47 | 3.46 |  | 32.17 | 36.17 | 2.58 |
| P 4 |  |  |  |  |  |  |  |  |  |  |  |  |
| d1 |  | 21.27 | 22.97 | 2.58 |  | 55.43 | 49.10 | 3.46 |  | 23.33 | 27.97 | 2.58 |
| d2 |  | 32.20 | 22.83 | 2.42 |  | 50.37 | 51.17 | 3.46 |  | 17.43 | 26.00 | 2.58 |
| d3 |  | 31.43 | 33.13 | 3.18 |  | 51.20 | 47.77 | 3.46 |  | 17.37 | 19.10 | 2.58 |
| P 5 |  |  |  |  |  |  |  |  |  |  |  |  |
| d1 |  | 40.13 | 47.7 | 2.58 |  | 54.77 | 48.23 | 3.46 |  | 5.10 | 4.07 | 2.58 |
| d2 |  | 45.23 | 50.0 | 2.42 |  | 51.93 | 48.30 | 3.46 |  | 2.80 | 1.73 | 2.58 |
| d3 |  | 45.23 | 57.80 | 3.18 |  | 51.33 | 41.07 | 3.46 |  | 3.43 | 1.17 | 2.58 |
| P 6 |  |  |  |  |  |  |  |  |  |  |  |  |
| d1 |  | 5.16 | 6.70 | 2.58 |  | 64.43 | 60.07 | 3.46 |  | 30.43 | 33.23 | 2.58 |
| d2 |  | 2.93 | 1.60 | 2.42 |  | 61.77 | 59.63 | 3.46 |  | 35.33 | 38.73 | 2.58 |
| d3 |  | 1.33 | 0.73 | 3.18 |  | 53.60 | 59.03 | 3.46 |  | 45.03 | 40.17 | 2.58 |

^1^ P: Period; d: day

^2^AH: Angus-Hereford;

^3^RC: Rarámuri Criollo

Table S6. Mean and standard error of motion index by cow, according to breed, period and day nested within period.

| Period/day^1^ | Angus-Hereford | Rarámuri Criollo | SE |
| --- | --- | --- | --- |
| P1 |  |  |  |
| d1 | 22386 | 17217 | 1455.4 |
| d2 | 22201 | 12337 | 1455.4 |
| d3 | 22463 | 11787 | 1455.4 |
| P 2 |  |  |  |
| d1 | 21263 | 11093 | 1455.4 |
| d2 | 21742 | 11766 | 1455.4 |
| d3 | 21088 | 11786 | 1455.4 |
| P 3 |  |  |  |
| d1 | 20296 | 10603 | 1455.4 |
| d2 | 19130 | 10469 | 1455.4 |
| d3 | 17713 | 11121 | 1455.4 |
| P 4 |  |  |  |
| d1 | 26118 | 12536 | 1455.4 |
| d2 | 24060 | 12984 | 1455.4 |
| d3 | 22923 | 12305 | 1455.4 |
| P 5 |  |  |  |
| d1 | 21207 | 10315 | 1455.4 |
| d2 | 20477 | 11005 | 1455.4 |
| d3 | 19368 | 9998 | 1455.4 |
| P 6 |  |  |  |
| d1 | 20394 | 11302 | 1455.4 |
| d2 | 18950 | 11237 | 1455.4 |
| d3 | 19192 | 11320 | 1455.4 |

^1^ P: Period; d: day

Table S7. Mean and SE for initial and final body weight and body condition score of Angus-Hereford and Rarámuri Criollo cows trained with virtual fencing collars.

| Breed | Body weight | | | |  | Body condition score | | | |
| --- | --- | --- | --- | --- | --- | --- | --- | --- | --- |
|  | Initial | Final | SE | *P*-value |  | Initial | Final | SE | *P*-value |
| Angus-Hereford | 442.0 | 466.3 | 8.5 | 0.104 |  | 5.7 | 5.7 | 0.15 | 0.771 |
| Rarámuri Criollo | 365.5 | 365.3 | 3.5 | 0.980 |  | 5.3 | 5.7 | 0.08 | 0.028 |
